# Endoplasmic Reticulum and Mitochondria Contacts Correlate with the Presence and Severity of NASH in Humans

**DOI:** 10.3390/ijms23158348

**Published:** 2022-07-28

**Authors:** Chaonan Jin, Eric Felli, Naomi Franziska Lange, Annalisa Berzigotti, Jordi Gracia-Sancho, Jean-François Dufour

**Affiliations:** 1 Department of Visceral Surgery and Medicine, Inselspital, Bern University Hospital, University of Bern, 3012 Bern, Switzerland; chaonan.jin@dbmr.unibe.ch (C.J.); eric.felli@dbmr.unibe.ch (E.F.); naomifranziska.lange@insel.ch (N.F.L.); annalisa.berzigotti@insel.ch (A.B.); 2 Department for BioMedical Research, Hepatology, University of Bern, 3012 Bern, Switzerland; 3 Graduate School for Cellular and Biomedical Sciences, University of Bern, 3012 Bern, Switzerland; 4 Graduate School for Health Sciences, University of Bern, 3012 Bern, Switzerland; 5 Liver Vascular Biology Research Group, CIBEREHD, IDIBAPS Research Institute, 08036 Barcelona, Spain

**Keywords:** mitochondria, endoplasmic reticulum, NASH, NAFLD, MAFLD

## Abstract

The interaction between the mitochondria and the endoplasmic reticulum (ER) is essential for hepatocyte function. An increase in ER–mitochondria contacts (ERMCs) is associated with various metabolic diseases. Non-alcoholic fatty liver disease (NAFLD) is associated with obesity and type 2 diabetes, and its progressive form non-alcoholic steatohepatitis (NASH) can lead to cirrhosis and hepatocellular carcinoma. However, the role of ERMCs in the progression of NAFL to NASH is still unclear. We assessed whether ERMCs could correlate with NAFLD severity. We used a proximity ligation assay to measure the abundance of ERMCs in liver biopsies from patients with biopsy-proven NAFLD (*n* = 48) and correlated the results with histological and metabolic syndrome (MetS) features. NAFLD patients were included according to inclusion and exclusion criteria, and then assigned to NAFL (*n* = 9) and NASH (*n* = 39) groups. ERMCs density could discriminate NASH from NAFL (sensitivity 61.5%, specificity 100%). ERMCs abundance correlated with hepatocellular ballooning. Moreover, the density of ERMCs increased with an increase in the number of MetS features. In conclusion, ERMCs increased from NAFL to NASH, in parallel with the number of MetS features, supporting a role for this interaction in the pathophysiology of NASH.

## 1. Introduction

In the last decade, the dynamic crosstalk between mitochondria and endoplasmic reticulum (ER) has gained attention given their correlation in metabolic syndrome (MetS) features such as insulin resistance and obesity [1,2,3]. Several features of MetS, most notably obesity, insulin resistance, and type 2 diabetes mellitus, have been implicated to contribute to non-alcoholic fatty liver (NAFL) progression. This stimulated the introduction of a novel nomenclature known as metabolic (dysfunction)-associated fatty liver disease (MAFLD) [4]. However, understanding the correlation between the mitochondria and the ER in MetS with MAFLD progression, from NAFL to non-alcoholic steatohepatitis (NASH), is still challenging [5].

When in proximity, the ER membrane and the mitochondrial outer membrane allow for the exchange of metabolites, including Ca^2+^, at specific sites called ER–mitochondria contacts (ERMCs). The structures involved in ERMCs are stabilized by strings of interacting mitochondrial and ER proteins, mainly including the inositol-1,4,5-trisphosphate receptor (IP_3_R) on the ER membrane, and the voltage-dependent anion channel (VDAC) on the mitochondrial outer membrane. These are coupled by the stress-inducible chaperone, glucose-regulated protein 75 (Grp75 or mortalin). Ca^2+^ is transported into the mitochondrial matrix via the mitochondrial calcium uniporter (mtCU) channel. The proximity of the above-mentioned Ca^2+^ channels allows ion signaling to efficiently pass from the ER lumen (higher Ca^2+^ concentration), into the mitochondrial matrix. Here, this flow regulates important cell functions, including the control of insulin signaling in hepatocytes.

Preclinical studies have reported that organelle interactions are disrupted in insulin-resistant tissues of several mouse models of obesity and type 2 diabetes [2,6,7]. Moreover, the disruption of Ca^2+^ transfer is associated with ER stress, mitochondrial dysfunction, lipid accumulation, activation of c-Jun N-terminal kinase and protein kinase Cε, and insulin resistance [6]. Interestingly, the fasted-to-postprandial transition also reduces the number of ER–mitochondria contact points in mouse livers [8].

Considering this background, we hypothesized that hepatic ERMCs increase with the severity of MetS features and over the NAFLD spectrum. In the present observational study, we aimed to quantify the IP3R1–VDAC1 interaction as a key functional ERMC for Ca^2+^ transport in patients covering the spectrum of NAFLD.

## 2. Results

### 2.1. Patient Characteristics

Forty-eight patients (9 NAFL and 39 NASH) were included in the analysis. The main demographic and clinical features of NAFL and NASH patients are presented in Table 1. In comparison to patients with NAFL, NASH patients tended to be older, although the difference was not significant. In both groups, most patients were male. Although all values were numerically higher in NASH patients, there were no significant differences in body mass index (BMI) and the presence of comorbidities (including obesity, type 2 diabetes (T2D), hypertension, and dyslipidemia). Biomedical parameters showed no statistically significant differences between the NAFL and NASH groups, except for prothrombin time (PT) and serum total bilirubin level. As expected, all NASH patients had higher grades of steatosis, ballooning, lobular inflammation, fibrosis, and overall SAF activity score. 

### 2.2. Analysis of ER–Mitochondria Contacts

#### 2.2.1. NAFL vs. NASH

**Figure 1:**
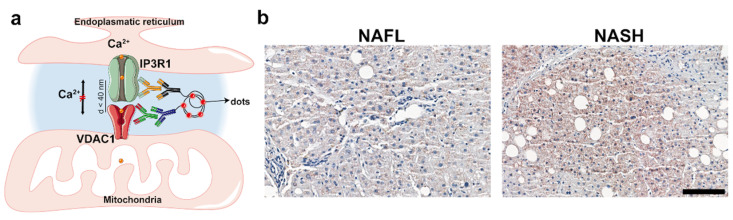
Schematic diagram of the PLA and representative images from NAFL and NASH liver biopsies. (**a**) Schematic diagram of the PLA; (**b**) representative images taken from 3dhistech caseviewer under 20× magnification (scale bars: 100 μm).

**Figure 2:**
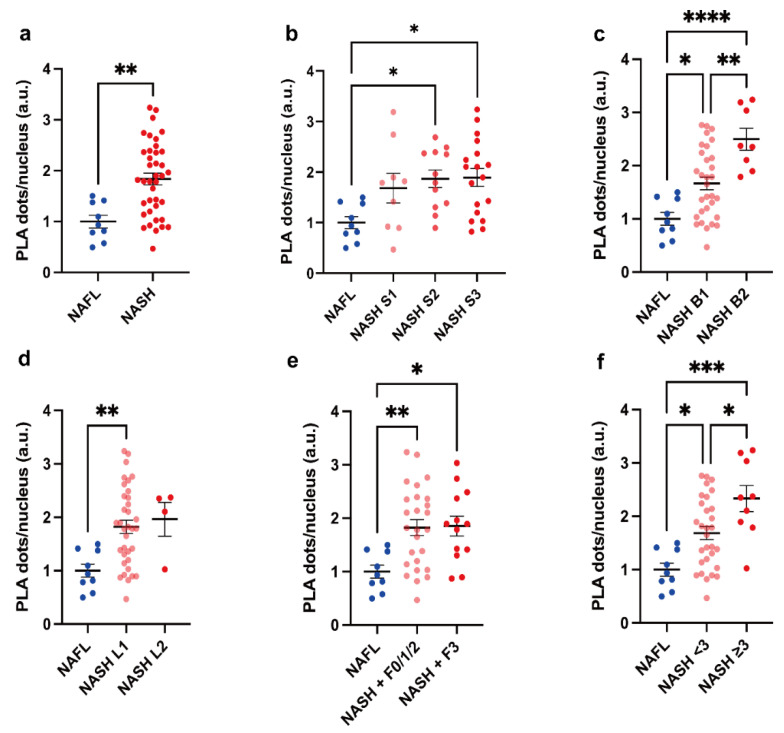
Comparison of PLA-dots/cell between PLA-dots/cell by NAFL and grades of NASH according to histology. (**a**) Comparison of PLA-dots/nucleus in NAFL (*n* = 9) and NASH (*n* = 39), (**b**) steatosis (NASH S1, S2, S3: *n* = 9, 12, 18, respectively), (**c**) ballooning (NASH B1, B2: *n* = 31, 8, respectively), (**d**) lobular inflammation (NASH L1, L2: *n* = 35, 4, respectively), (**e**) fibrosis (NASH F0/1/2, F3: *n* = 26, 13, respectively), and (**f**) SAF activity (NASH SAF activity score <3, ≥3: *n* = 30, 9, respectively). Results were analyzed by Student’s *t*-test or one-way ANOVA with Tukey’s multiple comparison test. A two-tailed *p*-value < 0.05 was considered significant. All statistical tests were two-sided. * *p* < 0.05, ** *p* < 0.01, *** *p* < 0.001, **** *p* < 0.0001. Data are expressed as mean ± SEM.

The quantification of ERMCs (PLA dots per nucleus) in the NAFL and NASH groups revealed that their abundance was significantly higher in NASH patients than in NAFL patients (*p* = 0.0017) (Figure 1a,b and Figure 2a).

To further explore if there was an increasing trend from NAFL to different severity stages of NASH, NASH subjects were categorized according to histological criteria of disease severity and comparisons were performed among groups (Figure 2a–f). ERMCs were significantly higher in grade 2 and 3 steatosis, grade 1 and 2 ballooning, grade 1 lobular inflammation, and grade 2 and 3 fibrosis in NASH compared to in NAFL. There was a substantial difference between NASH patients with ballooning grade 1 and 2, as well as a trend for increased values of the SAF activity score from NAFL, non-severe NASH, to severe NASH, defined by an activity score above 3 in the SAF scoring system; all *p* < 0.05.

Additional analysis of threshold levels of PLA-dots/cell indicated a significant discrimination between NASH and NAFL (ROC = 0.8348, *p* = 0.0019, *n* = 48) (Appendix A). We optimized the threshold analysis by Youden index. Applying the identified cut-off value of 21.9 (PLA-dots/cell) resulted in a sensitivity of 61.5% and specificity of 100% in differentiating NASH from NAFL.

#### 2.2.2. Metabolic Syndrome Contributions

**Figure 3:**
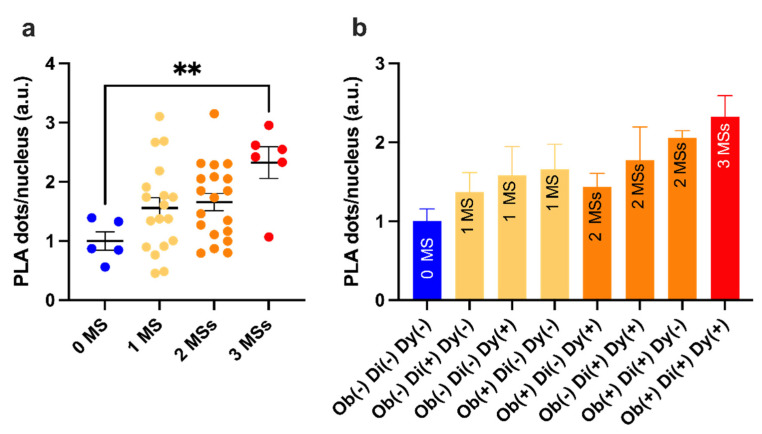
PLA-dots/cell and metabolic syndrome contributions. (**a**) ERMC data were grouped and analyzed depending on the number of MetS features that had a contribution. 0MS, *n* = 5; 1 MS, *n* = 18; 2 MS, *n* = 19; 3 MS, *n* = 6. (**b**) A more detailed analysis with the histogram reports the mean and SEM of the ERMC in NAFLD patients with different combinations of MetS features, showing an increasing trend from NAFLD with no MetS features to NAFLD with all three MetS features. Abbreviations: Di, diabetes mellitus type 2; Dy, dyslipidemia; MetS, metabolic syndrome; Ob, obesity. Ob(+)Di(+)Dy(+), *n* = 6; Ob(+)Di(+)Dy(−), *n* = 4; Di(+)Dy(+)Ob(−), *n* = 5; Ob(+)Dy(+)Di(−), *n* = 10; Ob(+)Di(−)Dy(−), *n* = 8; Dy(+)Ob(−)Di(−), *n* = 5; Di(+)Ob(−)Dy(−), *n* = 5; Di(−)Dy(−)Ob(−), *n* = 5. Results were analyzed by one-way ANOVA with Dunnett’s multiple comparisons test. All data were normalized to the “0 MetS” control group. A two-tailed *p*-value < 0.05 was considered significant. All statistical tests were two-sided. ** *p* < 0.01. Data are expressed as mean ± SEM.

MetS features were evaluated by clustering ERMCs data (Figure 3a). Although 1 and 2 MetS features did not result in a statistically significant increase (*p* = 0.21, *p* = 0.12, respectively), it is worth highlighting that the increase was ~1.5-fold higher than the control (0 MetS) (1.5 ± 0.2 1 MetS feature, and 1.7 ± 0.2 2 MetS features, respectively). Furthermore, a significant increase was found with 3 MetS features group when compared with the control; the respective fold change was 2.32 ± 0.27 higher (*p* = 0.0054). A more detailed histogram incorporating obesity, T2D, and dyslipidemia in NAFLD showed the different ERMCs levels in NAFLD patients with various combinations of MetS status (Figure 3b). This highlights an upward trend in ERMCs levels in NAFLD patients depending on additional MetS features.

**Figure 4:**
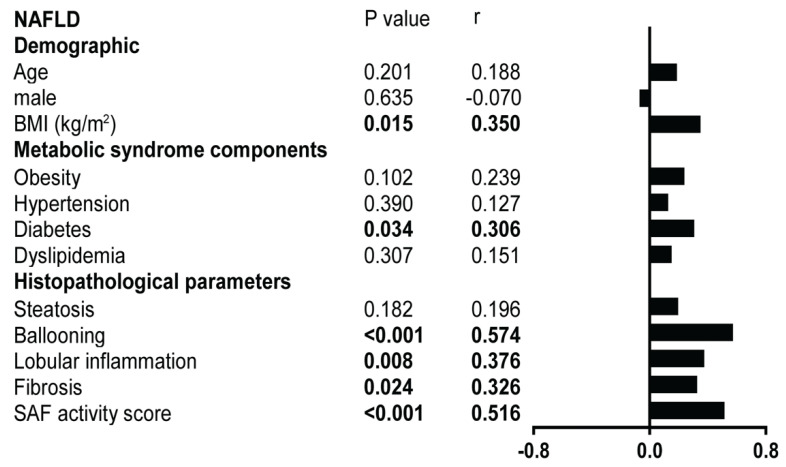
Correlation with demographic, metabolic syndrome features, and histopathological features in NAFLD (*n* = 48). The left panel presents Pearson correlation coefficient *r* and *p*-values for each variable. The right panel/vertical histogram shows different r levels for each variable with PLA dots/cell. SAF scores were adopted for histological parameters.

We observed a positive correlation between ERMCs and ballooning and SAF activity score in NAFLD. The steatosis grade did not correlate with ERMCs (Figure 4).

## 3. Discussion

A quarter of adults worldwide have been diagnosed with NAFLD; hence, there is a need for better understanding its progressive form NASH [9]. The recent discussion for the introduction of the MAFLD nomenclature was driven by the need to consider MetS contribution in NAFLD and aimed to clarify the mechanism of NAFLD progression to NASH. MAFLD is characterized by hepatic steatosis along with signs of metabolic dysregulation, including type 2 diabetes and obesity [4]. Similarly, ERMCs are associated with MetS such as insulin resistance and obesity [10,11]. We hypothesized that the altered crosstalk between the ER and mitochondria could be one of the pathophysiological features of MAFLD and NAFLD. 

In our study, we observed that hepatic ERMCs increased across the spectrum of NAFLD together with the severity of NASH and to the MetS features. Moreover, we reported increased hepatic ERMCs in the presence of obesity, T2D, and dyslipidemia in NAFLD. This supports previous observations that chronically increased IP3R1-mediated Ca^2+^ signaling led to excessive ERMCs and mitochondrial dysfunction, while reduced IP3R1 expression corrected mitochondrial dysfunction in high-fat-diet–fed mice [1,12]. Liver biopsies for our study were conducted in fasting conditions. Future studies assessing ERMCs in pre-clinical models of NAFLD with MetS will help to clarify these controversies and to investigate the underlying mechanisms and possible therapeutic opportunities. 

A previous study by Feriod et al., reported that the hepatic expression of IP3R1 mRNA and the extent of ER–mitochondria co-localization in human liver tissue were higher in NASH compared to NAFL [12]. This was confirmed by the increasing trend of ERMCs along the spectrum from NAFL to severe NASH in our study. While Nathanson et al., used a non-functional protein pair, Tom22 (located in the outer mitochondrial membrane) and protein disulfide isomerase (located inside the ER) to estimate ERMCs, we directly analyzed organelle interactions using PLA. We found that increased organelle contacts were associated with advanced histological features including ballooning, inflammation, fibrosis, and SAF activity score in NAFLD. The strong association with hepatocyte ballooning, the histologic hallmark of cellular injury and death, in NASH [13], further supports the importance of ERMCs in NASH pathogenesis.

We acknowledge limitations including the small sample size and a lack of diabetic and obese subjects without NAFLD for comparison. Longitudinal studies will be needed to further characterize the role of ER–mitochondria interactions in the progression of liver disease in NAFLD patients. Nevertheless, to the best of our knowledge, this is the first study to quantify hepatic ERMCs across the spectrum of NAFLD in relation to metabolic syndrome components and histological features of NAFLD in human samples. Finally, although our findings may improve the understanding of NASH development, caution should be applied when generalizing its conclusions due to the low variability of patient characteristics (i.e., racial homogeneity, presence of MetS).

In conclusion, NAFLD histological features, including ballooning, inflammation, fibrosis, and SAF activity score, correlate with the abundance of ERMCs in human hepatocytes. Furthermore, ERMCs quantitation allows the discrimination between NASH and NAFL, pointing to a potential use as a quantitative diagnostic biomarker in liver biopsy specimens. Increased ER–mitochondria interaction, as indicated by increased ERMCs, may be involved in the pathogenesis of NAFLD progression.

## 4. Materials and Methods

### 4.1. Study Design

NAFLD patients were included according to inclusion and exclusion criteria, then assigned to NAFL (*n* = 9) and NASH (*n* = 39) groups, with corresponding liver biopsy samples and clinical and histological data collected. Proximity ligation assay was employed to evaluate the abundance of ERMCs in liver biopsies from patients. Correlations between ERMCs and histological/MetS features were calculated. 

### 4.2. Population of the Study

Six-hundred and thirty-two patients who underwent a liver biopsy and were identified from the electronic patient records of the liver unit of the University Hospital Bern were selected, and then screened for eligibility between 1 January 2017 and 1 November 2019. One-hundred and eighty-seven patients underwent liver biopsy for suspicion of NAFLD/NASH and were evaluated for eligibility. Seventy-seven patients were included in the study. The 77 patients fulfilled the following inclusion criteria in the present study: (1) liver biopsy performed for suspicion of NAFLD [14]; (2) aged ≥18 years; (3) provided consent to the reuse of their health-related data; (4) remnants of liver biopsy available for analysis. We then excluded patients if any of the following were present at time of biopsy: (1) use of steatosis-inducing drugs (methotrexate; amiodarone; tamoxifen; systemic corticotherapy); (2) excessive alcohol consumption (210 g/week in men and >140 g/week in women); (3) other causes of chronic liver disease (chronic viral hepatitis B or C; hemochromatosis; autoimmune hepatitis; cholestatic liver diseases; alpha-1 antitrypsin deficiency; Wilson disease); (4) decompensated cirrhosis (encephalopathy; variceal bleeding; liver failure; ascites); (5) systematic infection; (6) hepatocellular carcinoma. We further excluded patients in whom (7) the period between blood sample and liver biopsy exceeded 6 months, and (8) no remnant tissue from the index liver biopsy was available for analysis. One patient was excluded because fewer than 6 random selected images were available. Finally, 48 patients were included in the analysis. This study was approved by the Cantonal Ethical Committee of Bern (KEK number 2020-02756). A general consent was signed by all participants.

### 4.3. Data Extraction

Clinical data and blood samples were collected during the routine patient visit to the hepatology outpatient clinic after an overnight fast. All samples were taken within 6 months of liver biopsy. 

### 4.4. Histological Assessment

Histological assessment of NAFLD and NASH samples was made according to the steatosis, activity, and fibrosis (SAF) classification [15] by an experienced liver pathologist who was blinded to clinical data and study design. The average length of biopsies was 23.89 ± 1.33 mm with 13 ± 0.6 portal tracts per patient. Histologically severe disease was defined as SAF activity score of ≥3 and/or fibrosis stage ≥3 [16]. 

### 4.5. In Situ Proximity Ligation Assay

To evaluate the association of the linked protein pair (IP3R1 and VDAC1) as an indicator of ERMCs, we applied proximity ligation assay (PLA) on processed paraffin-embedded slides from NASH and NAFL liver biopsies (Figure 1a). PLA is an antibody-based method to identify proteins at a distance of <40 nm. We conducted in situ staining of IP3R1-VDAC1 contacts following the Duolink PLA with Brightfield Protocol (Sigma-Aldrich, St. Louis, MO, USA). 

Liver sections were deparaffinized with xylene and dehydrated through a graded ethanol series, followed by antigen retrieval (sodium citrate buffer: pH 6.0) and washing with wash buffer for Brightfield. After blocking, slides were incubated overnight at 4 °C with primary antibodies against IP3R1 (Abcam (Cambridge, UK) 5804, 1:2000) and VDAC1 (Abcam 14,734, 1:8000). Then, the corresponding PLUS (Duolink In Situ PLA probe anti-Mouse PLUS, DUO92001) and MINUS (Duolink In Situ PLA probe anti-Rabbit MINUS, DUO92005) probes, each containing a short sequence of complementary oligonucleotides, were combined with antibodies. The two DNA tags were ligated with each other to form a DNA circle (ligation DUO82009 and ligase DUO82027) and further amplified using amplification enzymes (amplification DUO82050 and polymerase DUO82028). Next, detection solutions (DUO82051), substrate reagents (Substrate Reagent A DUO82055, Substrate Reagent B DUO82056, Substrate Reagent C DUO82057, Substrate Reagent D DUO82058), nuclear staining (DUO82059), mounting media, and coverslips were applied according to protocol.

After processing, slides were scanned using a PANNORAMIC Digital Slide Scanner under 20× magnification. For analysis, six random fields of view under 40× magnification were selected, and images were taken from Caseviewer software (3DHISTECH, Budapest, Hungary). Fiji-ImageJ software (2.1.0/1.53c) was used to quantify PLA signals (shown as brown dots). Nuclei in the respective fields were counted manually. Images were taken randomly and quantified in a blind fashion, with unblinding performed once all data were recorded. Results were expressed as dots per nucleus. 

### 4.6. Statistical Analysis

Statistical analyses were performed using SPSS 25.0 (IBM, Armonk, NY, USA) software and GraphPad Prism 9. A two-tailed Mann–Whitney U Test or a Student’s unpaired *t*-test was used for continuous variables in two groups, χ^2^ analysis or Fisher’s exact test for categorical variables. Continuous variables were expressed as mean ± standard deviation (SD), standard error of mean (SEM), or median and interquartile range (IQR), as appropriate. Categorical variables were reported as counts (percentage). Pearson’s test was used for calculating correlation. Receiver operating characteristic (ROC) curve analysis was performed. The Youden index was used to identify optimal cut-off values as previously described [17]. A two-tailed *p*-value of < 0.05 was regarded as significant.

## Figures and Tables

**Table 1:** Baseline information of study population. The bold value indicates all the significant *p*-values < 0.05.

	NAFL (*n* = 9)	NASH (*n* = 39)	*p*-Value
**Demographic**			
Age, years *	38.7–58.8	50.2–66.8	0.075
Male, *n* (%) **	7 (77.8)	27 (69.2)	0.611
BMI (kg/m^2^) *	27.9–30.8	29.1–34.6	0.129
**Comorbidities, *n* (%)**			
Obesity **	3 (33.3)	25 (64.1)	0.091
Hypertension **	7 (77.8)	30 (76.9)	0.956
T2D **	3 (33.3)	17 (43.6)	0.574
Dyslipidemia **	6 (66.7)	20 (51.3)	0.404
**Biomedical parameters**			
Glycemia (mmol/L) *	4.8–6.6	4.9–6.4	0.593
Creatinine (µmol/L) ***	82.3 ± 14.2	76.9 ± 15.0	0.330
Albumin (g/L) ***	40.1 ± 3.7	37.8 ± 3.8	0.134
AST (IU/L) *	30–60	40–73.5	0.130
ALT (IU/L) ***	72.3 ± 36.1	70.9 ± 39.8	0.756
Total bilirubin (µmol/L) *	10.0 (8.0–12.5)	13.0 (10.0–18.5)	0.047
ALP (IU/L) ***	88.2 ± 62.8	90.9 ± 27.1	0.844
GGT (IU/L) *	39–104.5	58.5–193.0	0.170
Total cholesterol (mmol/L) ***	5.0 ± 0.953	4.5 ± 1.075	0.288
HDL cholesterol (mmol/L) ***	1.2 ± 0.4	1.1 ± 0.3	0.448
LDL cholesterol (mmol/L) ***	2.6 ± 0.5	2.6 ± 1.0	0.961
Triglycerides (mmol/L) *	1.2–3.1	1.2–1.8	0.335
HbA1c (%) *	5.2–6.2	5.3–6.2	0.369
Platelets (G/L) ***	249.6 ± 50.5	209.5 ± 75.1	0.136
INR *	1–1.03	1–1.06	0.158
PT (s) *	10–11.0	10.7–11.9	0.008
Insulin (µU/mL) *	10.6–28.5	16.2–32.7	0.333
**Histological**			
**Steatosis grade, *n* (%)**			0.032
Steatosis 1 **	6 (66.67)	9 (23.08)	
Steatosis 2 **	2 (22.22)	12 (30.77)	
Steatosis 3 **	1 (11.11)	18 (46.15)	
**Ballooning grade, *n* (%)**			<0.001
Ballooning 0 **	6 (66.67)	0 (00.00)	
Ballooning 1 **	3 (33.33)	31 (79.49)	
Ballooning 2 **	0 (00.00)	8 (20.51)	
**Lobular inflammation grade, *n* (%)**			<0.001
Lobular inflammation 0 **	8 (88.89)	1 (2.56)	
Lobular inflammation 1 **	1 (11.11)	34 (87.18)	
Lobular inflammation 2 **	0 (00.00)	4 (10.26)	
**Fibrosis grade, *n* (%)**			<0.001
Fibrosis 0 **	5 (55.56)	2 (5.13)	
Fibrosis 1 **	2 (22.22)	9 (23.08)	
Fibrosis 2 **	2 (22.22)	15 (38.46)	
Fibrosis 3 **	0 (00.00)	13 (33.33)	
**SAF activity score** **as continuous variable**	0–1	2–4	<0.001
**SAF activity score, *n* (%)**			<0.001
SAF activity score 0 **	5 (55.56)	0 (00.00)	
SAF activity score 1 **	4 (44.44)	0 (00.00)	
SAF activity score 2 **	0 (00.00)	30 (76.92)	
SAF activity score 3 **	0 (00.00)	6 (15.38)	
SAF activity score 4 **	0 (00.00)	3 (7.69)	
	NAFL (*n* = 9)	NASH (*n* = 39)	*p*-value

BMI, body mass index; T2D, type 2 diabetes; AST, aspartate aminotransferase; ALT, alanine aminotransferase; ALP, alkaline phosphatase; GGT, gamma-glutamyltransferase; HDL, high-density lipoprotein; LDL, low-density lipoprotein; HbA1c, hemoglobin A1c; INR, international normalized ratio; PT, prothrombin time; SAF, steatosis, activity, and fibrosis. A two-tailed Mann–Whitney U Test or a Student’s unpaired *t*-test was used for continuous variables in two groups, χ^2^ analysis or Fisher’s exact test was used for categorical variables. Continuous variables were expressed as mean ± SD or median and IQR, as appropriate. Categorical variables were reported as counts (percentage). A two-tailed *p*-value of < 0.05 was regarded as significant. (*) interquartile range, (**) percentage, (***) mean ± SD.

**Figure A1:**
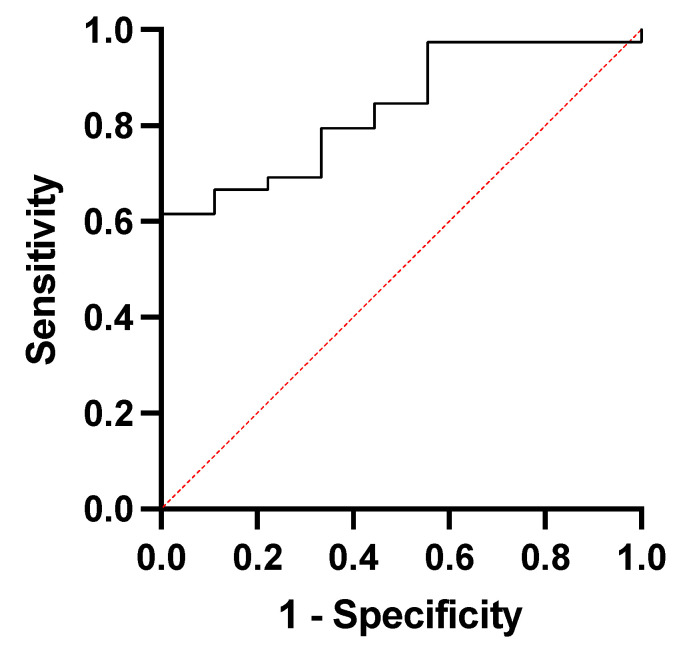
The ROC curve for differentiating ERMCs via PLA in NASH and NAFL.

## Data Availability

Not applicable.

## Appendix A

**Table A1:** The diagnosing performance of Figure A1 of PLA assay for NASH from NAFL.

**Area under the ROC Curve**	
Area	0.8348
Std. Error	0.06255
95% Confidence Interval	0.7122 to 0.9573
***p*** **Value**	0.0019
***p*** **Value Summary**	***

## References

[1] Arruda A.P., Pers B.M., Parlakgül G., Güney E., Inouye K., Hotamisligil G.S. (2014). Chronic enrichment of hepatic endoplasmic reticulum-mitochondria contact leads to mitochondrial dysfunction in obesity. Nat. Med..

[2] Tubbs E., Theurey P., Vial G., Bendridi N., Bravard A., Chauvin M.A., Ji-Cao J., Zoulim F., Bartosch B., Ovize M. (2014). Mitochondria-associated endoplasmic reticulum membrane (MAM) integrity is required for insulin signaling and is implicated in hepatic insulin resistance. Diabetes.

[3] Wang Y., Li G., Goode J., Paz J.C., Ouyang K., Screaton R., Fischer W.H., Chen J., Tabas I., Montminy M. (2012). Inositol-1,4,5-trisphosphate receptor regulates hepatic gluconeogenesis in fasting and diabetes. Nature.

[4] Eslam M., Newsome P.N., Sarin S.K., Anstee Q.M., Targher G., Romero-Gomez M., Zelber-Sagi S., Wai-Sun Wong V., Dufour J.F., Schattenberg J.M. (2020). A new definition for metabolic dysfunction-associated fatty liver disease: An international expert consensus statement. J. Hepatol..

[5] Lindenmeyer C.C., McCullough A.J. (2018). The Natural History of Nonalcoholic Fatty Liver Disease-An Evolving View. Clin Liver Dis..

[6] Rieusset J., Fauconnier J., Paillard M., Belaidi E., Tubbs E., Chauvin M.A., Durand A., Bravard A., Teixeira G., Bartosch B. (2016). Disruption of calcium transfer from ER to mitochondria links alterations of mitochondria-associated ER membrane integrity to hepatic insulin resistance. Diabetologia.

[7] Tubbs E., Axelsson A.S., Vial G., Wollheim C.B., Rieusset J., Rosengren A.H. (2018). Sulforaphane improves disrupted ER-mitochondria interactions and suppresses exaggerated hepatic glucose production. Diabetes.

[8] Theurey P., Tubbs E., Vial G., Jacquemetton J., Bendridi N., Chauvin M.A., Alam M.R., Le Romancer M., Vidal H., Rieusset J. (2016). Mitochondria-associated endoplasmic reticulum membranes allow adaptation of mitochondrial metabolism to glucose availability in the liver. J. Mol. Cell Biol..

[9] Younossi Z.M., Koenig A.B., Abdelatif D., Fazel Y., Henry L., Wymer M. (2016). Global epidemiology of nonalcoholic fatty liver disease-Meta-analytic assessment of prevalence, incidence, and outcomes. Hepatology.

[10] Stacchiotti A., Favero G., Lavazza A., Garcia-Gomez R., Monsalve M., Rezzani R. (2018). Perspective: Mitochondria-ER Contacts in Metabolic Cellular Stress Assessed by Microscopy. Cells.

[11] Townsend L.K., Brunetta H.S., Mori M.A.S. (2020). Mitochondria-associated ER membranes in glucose homeostasis and insulin resistance. Am. J. Physiol. Endocrinol. Metab..

[12] Feriod C.N., Oliveira A.G., Guerra M.T., Nguyen L., Richards K.M., Jurczak M.J., Ruan H.B., Camporez J.P., Yang X., Shulman G.I. (2017). Hepatic Inositol 1,4,5 Trisphosphate Receptor Type 1 Mediates Fatty Liver. Hepatol. Commun..

[13] Loomba R., Friedman S.L., Shulman G.I. (2021). Mechanisms and disease consequences of nonalcoholic fatty liver disease. Cell.

[14] (2016). EASL-EASD-EASO Clinical Practice Guidelines for the management of non-alcoholic fatty liver disease. J. Hepatol..

[15] Bedossa P., Poitou C., Veyrie N., Bouillot J.L., Basdevant A., Paradis V., Tordjman J., Clement K. (2012). Histopathological algorithm and scoring system for evaluation of liver lesions in morbidly obese patients. Hepatology.

[16] Nascimbeni F., Bedossa P., Fedchuk L., Pais R., Charlotte F., Lebray P., Poynard T., Ratziu V. (2020). Clinical validation of the FLIP algorithm and the SAF score in patients with non-alcoholic fatty liver disease. J. Hepatol..

[17] Unal I. (2017). Defining an Optimal Cut-Point Value in ROC Analysis: An Alternative Approach. Comput. Math. Methods Med..

